# A randomized, double-blind, placebo-controlled trial investigating cholesterol-lowering effects and safety of yellow yeast rice in adults with mild to moderate hypercholesterolemia: A study protocol

**DOI:** 10.1097/MD.0000000000011634

**Published:** 2018-07-27

**Authors:** Sewon Jeong, Jaekyung Lee, Oran Kwon, Ji Won Kim, Bumjo Oh

**Affiliations:** a BiofoodCRO Co, Ltd, Seodaemun-gu; b Department of Internal Medicine, SMG-SNU Boramae Medical Centre, Dongjak-gu; c Department of Nutritional Science and Food Management, Ewha Womans University; d Biofood Network, Seodaemun-gu; e Department of Family Medicine, SMG-SNU Boramae Medical Center, Dongjak-gu, Seoul, Korea.

**Keywords:** biomarkers, functional food, high serum cholesterol, randomized controlled trial, treatment outcome

## Abstract

**Background:**

Elevated levels of blood lipids are well-documented risk factors for cardiovascular disease. For cardiovascular risk reduction, preventive strategies to lower blood cholesterol levels are essential, and these strategies include lifestyle modification and cholesterol-lowering agents. We aim to investigate the cholesterol-lowering effects and safety of yellow yeast rice in a randomized, controlled, double-blind, and parallel group study.

**Methods:**

Participants for this study will be selected based on the following inclusion criteria:

1. Voluntary agreement to participate and sign the written informed consent forms
2. Men and women (aged 20–45 years, premenopause)
3. Body mass index: 18 to 30 kg/m^2^
4. Fasting low-density lipoprotein cholesterol (LDL-C) levels: ≥130 mg/dL
5. Fasting triglyceride levels: <300 mg/dL

Participants are randomly allocated to the placebo or yellow-yeast-rice-treated group. Participants with mild to moderately elevated LDL-C levels will consume 1 pouch of yellow yeast rice powder (containing monacolin K) or placebo twice daily for 8 weeks. Next, the lipid profiles will be evaluated.

**Results:**

The number of participants required for this study is 68, and is currently recruiting participants. Participants are randomly assigned to control group and intervention group.

**Conclusion:**

This is the first human intervention study to investigate the cholesterol-lowering effects and safety of yellow yeast rice in adults with mild to moderate hypercholesterolemia. Also, this is a randomized, double-blind, placebo-controlled trial that considers confounders, such as dietary habits, lifestyle factors, and genetic factors.

## 1 Introduction

Cardiovascular diseases (CVDs), such as myocardial infarction and stroke, are among the most serious diseases in developed and developing countries. Unfortunately, the absolute number of deaths from CVD, 750,000 deaths/year, has not changed for the last 25 years.^[1]^ It is estimated that by the year 2020, CVD will be responsible for 36% of all-cause deaths and the leading cause of death worldwide.^[2]^ As the life habits of Koreans are changing to those of people in Western countries in terms of consumption of high-fat meals, the number of patients with hyperlipidemia increased from 455,000 in 2005 to approximately 920,000 in 2009. Recently, the number of adolescents (<20 years old) with hyperlipidemia has also increased. Therefore, there is a need for systematic management and measures to prevent hyperlipidemia. Hypercholesterolemia is a key risk factor for CVD, such as myocardial infarction, stroke, and arteriosclerosis, particularly in combination with chronic diseases, such as hypertension and diabetes.^[2]^ Cholesterol accumulates on the arterial wall to form plaques, which narrow and harden the blood vessels, causing atherosclerosis. Thus, modulation of blood cholesterol levels is important in early stages to prevent CVD.^[3,4]^

Statins are considered the most effective agents for improving blood lipid profiles to reduce the risk of atherosclerosis.^[5]^ Despite statins being generally well tolerated, their use could be restricted by adverse effects, including elevated hepatic enzyme levels, gastrointestinal symptoms, and elevated creatine phosphokinase levels, particularly in elderly patients with multiple comorbid diseases using high-dose statins or a combination of lipid-lowering agents.^[6]^ Therefore, it is of clinical significance to find effective and safer alternative therapies, including food-based functional ingredients, to minimize the undesirable side effects in patients with the early stage hypercholesterolemia.^[7–9]^

Yellow yeast rice is a standardized food-based ingredient, prepared by using rice with *Aspergillus terreus*, a yeast mould traditionally used for brewing alcohol in South Korea. It contains multiple components, including natural monacolin K (>1.5 mg/g), a well-known cholesterol synthesis inhibitor.^[10]^

The cholesterol-lowering effects of red yeast rice, a food-based functional ingredient containing naturally derived monacolin K, have been investigated in various studies.^[11]^ A recent systematic literature review and meta-analysis of red yeast rice showed that administration of red yeast rice at doses of 1.2 to 2.4 g/d (4.8–24 mg/d as monacolin K) for 6 to 24 weeks reduced low-density lipoprotein cholesterol (LDL-C) levels by 1.02 mmol/L, compared to those in the placebo group, without any serious adverse events.^[8]^ Additionally, the effects of red yeast rice were comparable to those of statins.^[12,13]^

Similar to red yeast rice, yellow yeast rice contains monacolin K and might exhibit cholesterol-lowering effects in hypercholesterolemia. A previous study showed that administration of yellow rice yeast to male Sprague Dawley rats for 4 weeks reduced serum total cholesterol, LDL-C levels, and the atherogenic index of plasma.^[10]^ However, the cholesterol-lowering effects of yellow yeast rice have not been evaluated in well-designed human intervention studies.

In this study, we mainly aim to evaluate the effectiveness and safety of yellow yeast rice by using a randomized, double-blind, placebo-controlled design, including adult patients with mild to moderate hypercholesterolemia. We will analyze not only clinical indicators of cholesterol levels, such as blood lipid profiles, but also gene expression of markers related to cholesterol metabolism in peripheral blood mononuclear cells (PBMCs). The effects of cholesterol, diet, and lifestyle, which may be confounding variables, will be investigated, and a genotyping analysis will be performed for determining the effects of genetic factors on the cholesterol-lowering effects of yellow yeast rice.

## 2 Methods and analysis

### 2.1 Study design

This study is a randomized, double-blind, placebo-controlled, parallel group study with 2 intervention arms for investigating the effectiveness and safety of yellow yeast rice in controlling blood cholesterol levels. After obtaining informed consents from all participants and completing a baseline evaluation, including a brief medical examination to determine eligibility, participants who meet the eligibility criteria will start a 2-week run-in period, in which they have to follow the guidelines for diet and lifestyle control. After the 2-week run-in period, participants will be randomized into either the test or placebo group.

**Figure 1:**
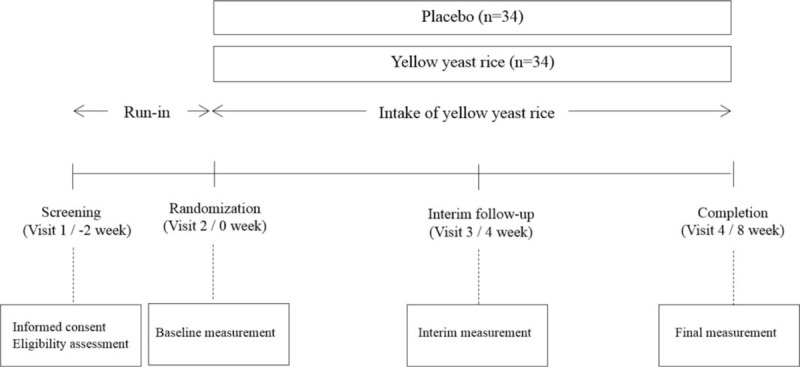
A schematic depiction of the study design.

Figure 1 shows a schematic depiction of the study design.

### 2.2 Study population and eligibility criteria

The study population is adults aged 20 to 45 years old. Assuming a 20% dropout rate, 68 eligible participants will be recruited from a single center, Seoul Metropolitan Government-Seoul National University Boramae Medical Centre.

Eligible participants are those who meet all the following inclusion criteria and none of the listed exclusion criteria.

*Inclusion criteria*:

1. Voluntary agreement to participate and sign the written informed consent forms
2. Men and women (aged 20–45 years, premenopause)
3. Body mass index (BMI) = 18 to 30 kg/m^2^
4. Fasting LDL-C levels: ≥130 mg/dL
5. Fasting triglyceride levels: <300 mg/dL

*Exclusion criteria*:

1. Continuous use of drugs that affect lipid metabolism or inflammation within 4 weeks before the first visit
2. Continuous ingestion of health functional foods (plant sterols, omega-3 fatty acids, aloe, chitosan, onion extract, etc) and oriental herbal medicines that affect lipid metabolism and inflammation within 4 weeks before the first visit
3. Weight loss therapy or weight change of more than 10% within 6 months before the first visit
4. Having any of the following diseases:
  - Myocardial infarction, stroke, or hypertension
  - Diagnosed diabetes or fasting blood glucose level >126 mg/dL at the first visit
  - Liver dysfunction
  - Renal impairment
  - Chronic inflammatory bowel disease
  - Autoimmune diseases, hyperthyroidism, hypothyroidism, or malignant tumors
5. Women receiving hormonal therapy
6. Excessive smokers (≥1 pack/d)
7. Alcoholics or consuming >140 g/wk
8. Regular intensive exercise (>10 h/wk)
9. Participation in other clinical studies within 4 weeks before the first visit
10. Allergy to yellow yeast rice
11. Pregnant or lactating women

### 2.3 Recruitment and screening

Participants are recruited using posters and advertisements in Seoul Metropolitan Government-Seoul National University Boramae Medical Centre and nearby areas. Posters and advertising descriptions contain brief introductions about this study and the contact information of the investigator.

Potential participants will receive the detailed information about this study and will have an informed discussion with trained research coordinators. Those who are interested in participating will have the screening assessment after signing informed consent forms. The effects of cholesterol, diet, and lifestyle, which may be confounding variables, will be investigated, and a genotyping analysis will be performed to determine the effects of genetic factors on the cholesterol-lowering effects of yellow yeast rice.

### 2.4 Randomization, allocation concealment, and blinding

Randomization sequence is computer generated by an independent statistician. All the labeled test and placebo products are provided by Daesang Co, Ltd (Seoul, South Korea). The randomization numbers are kept in opaque sealed envelopes. Group assignments are not revealed and are blinded to the participants and investigators throughout the entire study. The participants who meet the eligibility criteria are randomly assigned (1:1) to either receiving either placebo or yellow-yeast-rice-treated group. Randomization is stratified by gender. Allocation concealment is maintained since no sealed envelope is opened during the study.

### 2.5 Handling of withdrawal

Participants can withdraw their consents for any reason at any time. If any participant wishes to withdraw, investigators will ask about the reason for the withdrawal. Incidences of participant loss, follow-up and withdrawal, will be recorded and reported.

### 2.6 Intervention

#### 2.6.1 Test and control groups

Participants in the test group will receive yellow yeast rice powder (1 pouch) twice daily for 8 weeks. Yellow yeast rice powder contains 3.5 mg monacolin K/pouch (7 mg monacolin K/daily dose). Participants in the placebo group will receive rice power without monacolin K. Placebo and yellow yeast rice powder, both have the same color, taste, appearance, and flavor. Yellow yeast rice and placebo food, both are provided by Daesang Co. The packages of both yellow yeast rice and placebo will be dispensed and reclaimed by trained research coordinators. The principal investigator and research coordinator will be then responsible for storing and keeping records of the test and placebo products.

#### 2.6.2 Outcome measures

A summary of the schedule and all measurements is summarized in Table 1.

**Table 1:**
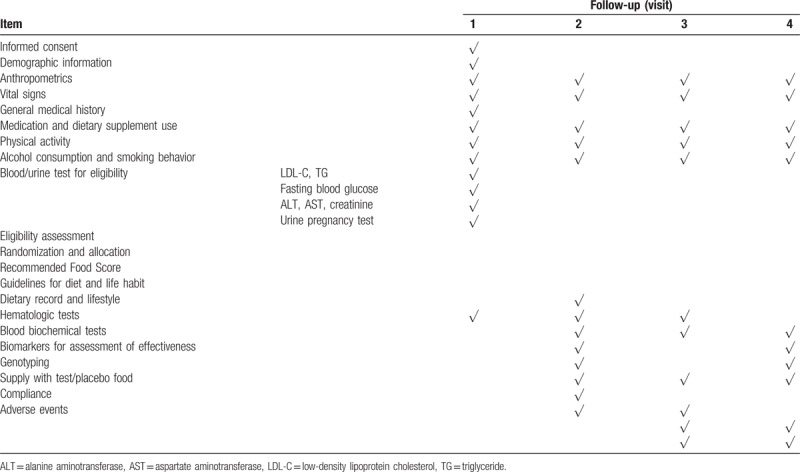
Schedule of data collection.

Outcomes related to effectiveness and safety, as well as possible covariables, including demographic information, anthropometric measures, dietary habits, physical activity, alcohol consumption, and smoking behavior, will be assessed. Face-to-face interviews will take place at the first screening, baseline, 4 weeks, and 8 weeks after intervention. Possible covariables will be surveyed using structured questionnaires.

Standardized interviews and blood sampling will be carried out by trained investigators and research coordinators. Participants will be instructed to arrive for laboratory testing between 8:30 am and 9:30 am after a 12-hour overnight fast.

##### 2.6.2.1 Effective variables

At baseline, 4, and 8 weeks after intervention, lipid profile, lipoproteins, glucose levels, and inflammation markers will be analyzed. Gene expression of markers related to cholesterol metabolism in PBMCs, such as 5-hydroxy-3-methylglutaryl-coenzyme A and LDL receptor, will be also analyzed using quantitative real-time polymerase chain reaction.

Brachial-ankle pulse wave velocity will be measured at baseline and 8 weeks after intervention. The measurements will be performed after the participant's rest for 15 minutes in the supine position.

##### 2.6.2.2 Safety variables

Hematologic tests, blood biochemical tests, and urine analysis will be performed at baseline and 8 weeks after intervention. At every visit, vital signs and adverse events will be monitored. Major indicators of vital signs include body temperature, systolic blood pressure, diastolic blood pressure, and pulse rate. Adverse events will be evaluated and classified during every visit throughout the intervention period. In addition, participants will be asked to report any adverse event they experience. All adverse events will be carefully monitored, and any unexpected symptom, abnormal vital sign, or discomfort will be recorded correspondingly. The principal investigator will evaluate all adverse events on a regular basis. If the adverse event cannot be resolved, follow-up will be continued until the adverse event disappears.

##### 2.6.2.3 Possible covariables

Age, gender, and general medical history will be assessed at baseline. Participants will not be allowed to receive concomitant interventions, such as drugs affecting lipid metabolism, including lipid-lowering agents, weight loss medications, antihypertensive agents, and oriental medicines. Continuous use of functional foods and ingredients, such as omega-3 fatty acids, plant sterols, psyllium seed husk, aloe, and chitosan, will not be allowed during the duration of the study. During the study period, current medication and supplement use by the participants will be assessed during every visit.

Weight, waist circumference, and BMI will be measured during every visit. Height will be measured in the standing position without shoes, and weight will be measured using a weighing scale with minimal clothes. BMI will be calculated by dividing the weight (in kilograms) by the square of the height (in meters). Waist circumference will be then measured in the standing position, intermediate between the lower margin of the ribs and the iliac crest. We will use the same equipment during each visit and will take special care so that the same researcher can measure these parameters at the same time.

Dietary intake and lifestyle, including habitual physical activity, history of alcohol consumption, and smoking, will be investigated. Normal dietary intake patterns will be assessed using the Recommended Food Score.^[14]^ Background dietary intake, including total energy and macronutrient, will be assessed using 3-day food intake records by a mobile application. Three-day food intake records will be collected for 2 weekdays and 1 weekend day at baseline, 4, and 8 weeks after intervention to monitor dietary compliance. Physical activity will be evaluated during screening using the International Physical Activity Questionnaire (IPAQ, Korean version).^[15]^ During enrolment, habitual physical activity levels will be assessed at baseline and during follow-up visits. During the study period, the participants will be asked to maintain their usual diets, smoking habits, and physical activity and asked to avoid excessive antioxidant-containing foods, such as vegetables and fruits. In addition, participants will not be allowed to consume alcohol or perform vigorous physical activity for 48 hours before investigator visits.

To evaluate the correlation between genetic factors and cholesterol-lowering effects of yellow yeast rice, polymorphism of genes related to cholesterol metabolism, such as *CETP* and *PCSK9,* will be determined at baseline levels.

#### 2.6.3 Compliance check

Compliance will be checked by counting the returned pouches. To stimulate compliance, participants will be contacted by telephone and reminded during follow-up visits.

#### 2.6.4 Power calculation

To detect changes in LDL-C levels, 27 participants per group will be selected, based on the results of a previous intervention study using red yeast rice, assuming a power of 80% and a 2-sided alpha level of 0.05.^[12,13]^ Assuming a 20% dropout rate, the sample size will be estimated at 34 participants per group.

### 2.7 Statistical analysis

Data analysis will be conducted by well-trained statisticians, not associated with the research team. Data will be examined for the presence of normality, outliers, violations, and/or missing data. A per-protocol analysis of the participants, who follow the protocol and complete the study, will be carried out. Baseline characteristics will be compared between groups using the Student *t* test. Continuous normally distributed variables will be compared between groups using analysis of covariance or linear mixed effect model. Pearson χ^2^ test or Fisher exact test will be used for categorical variables. For inter-group analysis, paired *t* test or Wilcoxon signed-rank test, will be performed for continuous variables. A *P*-value <.05 will be considered statistically significant. All analyses will be conducted using the SAS software. If needed, models will be adjusted for relevant confounding variables, and a stratified analysis will be performed.

### 2.8 Data collection and quality control

Data in this study will be compiled using the electronic case report forms. After every visit is completed at hospital, data will be entered using the electronic case report system. This study will be monitored by BiofoodCRO Co, Ltd (Seoul, South Korea). Periodic monitoring will allow high accuracy and quality throughout the study.

## 3 Results

**Figure 2:**
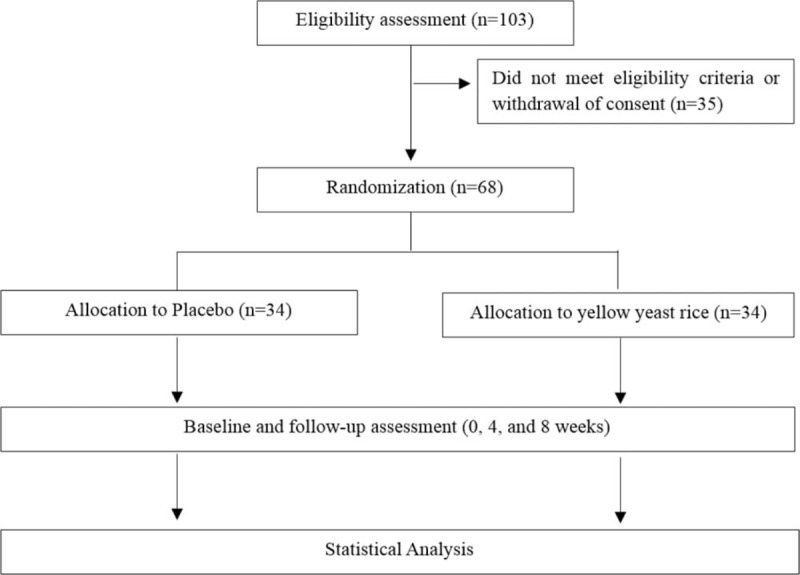
Consort diagram.

A consort diagram is described in Figure 2.

## 4 Discussion

To our knowledge, this is the first randomized, double-blind study to investigate the cholesterol-lowering effects of yellow yeast rice. We hope that the results of this study will provide scientific evidence on the effectiveness of yellow yeast rice against mild to moderate hypercholesterolemia in adult patients. To facilitate appropriate high-quality methodology and strict quality control, this protocol has been developed according to the CONSORT statement.^[16]^

## Author contributions

Sewon Jeong, Bumjo Oh, and Oran Kwon planned the study, designed the study protocol, and drafted the manuscript. Sewon Jeong wrote the manuscript, implemented the study, and finalized the study protocol. Bumjo Oh, Ji Won Kim, and Jaekyung Lee helped to develop the study measures and analyses. All authors contributed to the drafting of the manuscript and read and approved the final manuscript.

**Conceptualization:** Sewon Jeong, Oran Kwon, Ji Won Kim, Bumjo Oh.

**Data curation:** Jaekyung Lee, Bumjo Oh.

**Formal analysis:** Bumjo Oh.

**Funding acquisition:** Oran Kwon.

**Investigation:** Sewon Jeong, Oran Kwon, Bumjo Oh.

**Methodology:** Sewon Jeong, Bumjo Oh.

**Project administration:** Oran Kwon, Ji Won Kim, Bumjo Oh.

**Supervision:** Jaekyung Lee.

**Validation:** Bumjo Oh.

**Writing – original draft:** Sewon Jeong, Jaekyung Lee, Bumjo Oh.

**Writing – review & editing:** Oran Kwon, Ji Won Kim, Bumjo Oh.
